# Supplementary material for: GenPup-M: A novel validated owner-reported clinical metrology instrument for detecting early mobility changes in dogs
Source: PLoS One. 2023 Dec 27;18(12):e0291035. doi: 10.1371/journal.pone.0291035 (PMC10752556; doi:10.1371/journal.pone.0291035)
Supplement: S1 Fig — The clinical examination sheet was validated by Harris et al. (2018). The tool was used to assess joint function score (JFS) adapted by Impellizeri et al (2000) where ‘0’ represented the normal range of motion (ROM) and ‘4’ signified three to four joints abnormalities and a pain response was elicited upon touching the joint. Mobility/lameness scores in this validated clinical examination (0–10) were adapted from previous studies (Vasseur, 1993) to determine the dog’s ROM. Visual gait analysis can be conducted in the consultation room during history taking to reveal subtle lameness, including assessment of stand-up/lie-down phases (Hazewinkel, 2003). (PDF) [file pone.0291035.s001.pdf]

Supporting Information 1. The pre-validated veterinary clinical examination sheet used for this project. The clinical examination sheet was validated by Harris *et al*. (2018).

Date………….. Case no.: ……………

Clinical Checklist for Canine Osteoarthritis

Clinician: ………………………………………………….

Owner Name: ……………………………………………… Dog Name: ……………………………………………

1. General

   Age, breed, sex, neuter status:
   ………………………………………………………………………………………………………

   Weight and Body Condition Score (see attached key): …………………………………………………….

   OA history (brief summary; time of onset, signs of disease noticed by owner, analgesic drugs currently prescribed, when drugs were last taken etc.):
   ………
   ………
   ………
   ………
   ………

   Other health related issues (inc. **eye test**- blink reflex, tracking and obstacles)
   ………
   ………
   ………

2. Mobility

   A) Lameness during locomotion (0-10 NRS, See guidelines in Vasseur and Slatter 1993)

   - Overall Lameness score: ……….
   - Limbs affected: ……….

   B) Ability to:

   - Stand up: Score (0-3): …………………………….
   - Lie down: Score (0-3): …………………………….

3. Physical examination

Fore limbs

	Left		Right	
	☺	☹	☺	☹
Digital joints				
Carpus				
Elbow				
Shoulder				

Hind limbs

	Left		Right	
	☺	☹	☺	☹
Digital joints				
Tarsus				
Stifle				
Hip				

JFS and Abnormalities (see key for scores)

Joint Affected →										
Abnormalities (0/1)↓	L	R	L	R	L	R	L	R	L	R
Crepitus										
Tissue swelling										
Effusion										
Abnormal bone/ joint structure*										
Muscle atrophy**										
Inc. temperature										
JFS (0-4)										

5. Global Score

Overall judgement of severity of the animal's condition (tick correct)

	Owner	Clinician	Student
None			
Mild			
Moderate			
Severe			

Clinical Checklist key page 2

Lameness score (stand up/ lie down): 0= normal function, 1= quite slow/stiff, 2= very slow/stiff, 3= unable to perform action without assistance

Joint abnormality score

0= absent, 1= present

Joint function score (JFS) (adapted from Impellizeri 2000)

0- Normal range of motion, no stiffness evident and no pain response during manipulation
1- One of the following joint defects:
   Reduced range of motion
   Pain response to flexion
   Pain response to extension
   Pain response to other manipulation e.g. abduction (hip and shoulder), internal rotation (elbow), "drawer test" (stifle) etc.
2- Two of the joint defects described in score 1
3- Three/four of the joint defects described in score 1
4- Three/four of the joint defects described in score 1 **and** pain response to touching joint or limb/guarding limb.

>Pain response here may refer to vocalisation (e.g. yelp or whine), avoidance behaviour (e.g. struggling) aggressive behaviour, etc.

* e.g. Patella luxation, roughness of cortices etc

** (of associated limb)

The tool was used to assess joint function score (JFS) adapted by Impellizeri et al (2000) where '0' represented the normal range of motion (ROM) and '4' signified three to four joints abnormalities and a pain response was elicited upon touching the joint. Mobility/lameness scores in this validated clinical examination (0-10) were adapted from previous studies (Vasseur, 1993) to determine the dog's ROM. Visual gait analysis can be conducted in the consultation room during history taking to reveal subtle lameness, including assessment of stand-up/lie-down phases (Hazewinkel, 2003).
