# Supplementary material for: GenPup-M: A novel validated owner-reported clinical metrology instrument for detecting early mobility changes in dogs
Source: PLoS One. 2023 Dec 27;18(12):e0291035. doi: 10.1371/journal.pone.0291035 (PMC10752556; doi:10.1371/journal.pone.0291035)
Supplement: S2 Fig — GenPup-M can collect mobility data from dog owners at repeated time points and be used in clinical settings or within the Dogs Trust Generation Pup™ longitudinal study to identify risk factors indicative of early reductions in mobility. Hence, early interventions can be implemented, improving canine health and welfare. (PDF) [file pone.0291035.s002.pdf]

Supporting Information 2. The GenPup-M questionnaire has been developed as a mobility questionnaire which aims to accurately identify subtle changes relating to canine mobility.

‘Five month’ questionnaire

This questionnaire will allow us to find out about your dog's recent experiences with you. It is designed to be completed when your dog is approximately 5 months old.

Thank you in advance for your help with this survey.

Date when this questionnaire was completed

Insert date below…

____

Please write details below if you have had any changes in your contact details since the last questionnaire

Step 1 of 2...How is your puppy?

**1 How is your puppy in him/herself?**	
	Tick one box
Very well	☐
Fairly well	☐

Satisfactory	☐
Poorly	☐
Very poorly	☐

Step 2 of 2...Mobility and Exercise

Section 1 – Mobility

1 Does your puppy currently have ANY mobility problems, for example occasional stiffness when getting out of bed, difficulty jumping into the car/onto a bed or sofa?

I'm unsure	☐
Yes	☐
No	☐

1.a ****If you ticked 'I'm unsure' or 'No' in Q.1 please answer this question and then move Q.2****

Is your puppy currently receiving any medication/dietary supplements/other therapies or treatments to help him/her with his/her mobility?

	Tick one box
I'm unsure	☐
Yes	☐
No	☐

1.b ****If you ticked 'Yes' in Q.1 please answer this question, otherwise move to Q.3.a****

How old was "Toby when he started having his/her mobility problems?

	Tick one box
Not applicable - never had mobility problems	☐
1 month or less	☐
2 months	☐
3 months	☐
4 months	☐
5 months	☐
I don't know/can't remember	☐
Other (please specify) ____________________	☐

2 If you can, please list below any medications that your puppy is currently receiving for mobility problems.

3 If you can, please list below any medications that your puppy is currently receiving for problems other than mobility.

4 Please list any dietary supplements that your puppy is currently receiving.

5 Since I have owned him/her, my puppy has had…

Tick one box per row

	Never	Yes, in the past, but not currently	Yes, currently receiving	I don't know/can't remember
Acupuncture	☐	☐	☐	☐
Hydrotherapy	☐	☐	☐	☐
Physiotherapy	☐	☐	☐	☐
A massage/massages	☐	☐	☐	☐

6 Please use this space to tell us about any other treatments (for example stem cell therapy, laser, shock wave treatment) that you have used/are using to help your puppy with mobility problems…

____

____

____

____

____

____

____

____

Section 2 – Lifestyle

7 In the last week, on average, how far has your puppy exercised each day?

Tick one box

Less than a mile	☐
Between 1 mile and just under 2 miles	☐
Between 2 miles and just under 3 miles	☐
Between 3 miles and just under 4 miles	☐
4 or more miles	☐
I don't know/can't remember	☐
Other (please specify) ______________________	☐

8 Are there particular days of the week upon which your puppy has significantly more exercise?

	Tick all that apply
Mon	☐
Tues	☐
Weds	☐
Thurs	☐
Fri	☐
Sat	☐
Sun	☐

9 My puppy is exercised most often over the following sort of terrain…

	Tick a maximum of two
On level grass	☐
In woodland	☐
On roads/pavements	☐
Over rough hill ground	☐
On sand	☐
On pebbles	☐
Other (please specify) ______________________________	☐

10 At exercise, my puppy usually spends most of his/her time…

	Tick one box
Walking (on a long/flexi-lead)	☐
Walking (on a short lead)	☐
Walking (off lead, 'to heel')	☐
Walking (off lead, not 'to heel')	☐
Trotting (on a long/flexi-lead)	☐
Trotting (on a short lead)	☐
Trotting (off lead, 'to heel')	☐
Trotting (off lead, not 'to heel')	☐
Running freely (faster than a trot)	☐
Other (please specify) ______________________________	☐

**11 Does your puppy have his/her activity levels restricted when he/she is exercised?**	
	Tick one box
No - he/she can run around as much as he/she likes off lead	☐
Only if he/she looks tired and/or stiff/lame	☐
Yes - the walker limits his/her exercise	☐
Other (please specify) ______________________	☐

Section 3 – General Mobility

12 How much do you think your puppy's mobility adversely influences his/her general well being?

Please circle the answer for the question above…

Not applicable	*Not at all*					*Moderately*					*Extremely*
n/a	0	1	2	3	4	5	6	7	8	9	10

13 How disabled is your puppy by lameness?

Please circle the answer for the question above…

Not applicable	*Not at all*					*Moderately*					*Extremely*
n/a	0	1	2	3	4	5	6	7	8	9	10

14 How active is your puppy?

Please circle the answer for the question above…

Not applicable	*Extremely inactive*					*Moderately active*					*Extremely*
n/a	0	1	2	3	4	5	6	7	8	9	

**15 To what degree does your puppy show stiffness after a 'lie down'?**											
Please circle the answer for the question above…											
Not applicable	*Not at all*					*Moderately*					*Extremely*
n/a	0	1	2	3	4	5	6	7	8	9	10

Section 4 – Mobility at exercise

**16 How keen is your puppy to exercise?**											
Please circle the answer for the question above…											
Not applicable	*Not at all*					*Moderately*					*Extremely*
n/a	0	1	2	3	4	5	6	7	8	9	10

**17 How would you rate your puppy's ability to exercise?**											
Please circle the answer for the question above…											
Not applicable	*Extremely poor*					*Moderate*					*Extremely good*
n/a	0	1	2	3	4	5	6	7	8	9	10

**18 To what extent is your puppy's mobility adversely affected immediately after exercise?**											
Please circle the answer for the question above…											
Not applicable	*Not at all*					*Moderately*					*Extremely*
n/a	0	1	2	3	4	5	6	7	8	9	10

19 How often does your puppy rest (stop/sit down) during exercise?

Please circle the answer for the question above…

Not applicable	*Not at all*					*Moderately*					*Extremely frequently*
n/a	0	1	2	3	4	5	6	7	8	9	10

20 To what extent does cold, damp weather reduce your puppy's ability to exercise?

Please circle the answer for the question above…

Not applicable	*Not at all*					*Moderately*					*Ability to exercise extremely reduced*
n/a	0	1	2	3	4	5	6	7	8	9	10

21 To what extent does stiffness reduce your puppy's ability to exercise?

Please circle the answer for the question above…

Not applicable	*Not at all*					*Moderately*					*Ability to exercise extremely reduced*
n/a	0	1	2	3	4	5	6	7	8	9	10

22 Please use the space below to add any other information about your dog that you would like to share with us…

Please use the space below to share any other information with us…

Thank you so much for your help in completing this questionnaire.
Pleaseusethefreepostenvelopeprovidedtosendthisbacktous
at your earliest convenience!

GenPup-M can collect mobility data from dog owners at repeated time points and be used in clinical settings or within the Dogs Trust Generation Pup longitudinal study to identify risk factors indicative of early reductions in mobility. Hence, early interventions can be implemented, improving canine health and welfare.
