# Supplementary material for: GenPup-M: A novel validated owner-reported clinical metrology instrument for detecting early mobility changes in dogs
Source: PLoS One. 2023 Dec 27;18(12):e0291035. doi: 10.1371/journal.pone.0291035 (PMC10752556; doi:10.1371/journal.pone.0291035)
Supplement: S3 Fig — (PDF) [file pone.0291035.s003.pdf]

Supporting Information 3. Raw and normalised values for peak vertical forces (PVF) and absolute and relative speed

Peak Vertical Force

PID	Mobility Category	Peak Vertical Force (N/kg) (Thoracic)	Peak Vertical Force (N/kg) (Pelvic)
3	No Impairments	1.21	0.88
6	No Impairments	1.08	0.80
10	No Impairments	0.94	0.69
11	No Impairments	0.93	0.76
13	No Impairments	1.29	0.84
14	No Impairments	1.33	0.77
15	No Impairments	1.04	0.80
19	No Impairments	1.03	0.84
22	No Impairments	1.29	0.84
23	No Impairments	1.25	0.81
26	No Impairments	1.09	0.83
30	No Impairments	1.22	0.74
33	No Impairments	1.04	0.68
35	No Impairments	1.09	0.80
38	No Impairments	1.29	0.73
40	No Impairments	0.90	0.63
41	No Impairments	0.70	0.63
45	No Impairments	1.35	0.81
48	No Impairments	1.28	0.87
52	No Impairments	1.04	0.64
56	No Impairments	0.90	0.57
58	No Impairments	0.59	0.46
59	No Impairments	0.73	0.67
60	No Impairments	1.34	0.94
61	No Impairments	1.32	0.75
62	No Impairments	0.96	0.69
65	No Impairments	0.55	0.81
66	No Impairments	0.28	0.50
67	No Impairments	0.95	0.73
68	No Impairments	1.35	0.81
1	Combined	1.16	0.71
5	Pelvic	1.16	0.85
7	Thoracic	0.80	0.72
8	Combined	1.12	0.85
9	Combined	1.50	0.62
12	Pelvic	1.16	0.80

24	Pelvic	1.17	0.95
27	Pelvic	1.15	0.79
28	Pelvic	1.27	0.76
29	Pelvic	1.25	0.93
31	Combined	0.95	0.60
32	Thoracic	0.94	0.83
34	Combined	1.02	0.68
36	Thoracic	0.94	0.74
37	Thoracic	1.34	0.70
39	Pelvic	1.93	0.91
42	Pelvic	1.86	0.95
43	Pelvic	1.29	0.84
46	Thoracic	1.01	0.63
47	Pelvic	1.06	0.72
49	Pelvic	1.05	0.75
50	Combined	1.12	0.64
51	Thoracic	0.99	0.61
53	Pelvic	1.16	0.80
55	Thoracic	1.15	0.95
57	Thoracic	0.77	0.68
63	Pelvic	1.32	0.68
64	Pelvic	1.37	0.71

Absolute and Relative Speed

PID	Mobility Category	Average Speed (m/s)	Froude Number
3	No Impairments	2.20	0.72
6	No Impairments	1.78	0.62
10	No Impairments	1.92	0.77
11	No Impairments	2.08	0.74
13	No Impairments	1.74	0.94
14	No Impairments	1.83	0.74
15	No Impairments	1.93	0.71
19	No Impairments	1.98	1.05
22	No Impairments	1.53	0.81
23	No Impairments	1.70	0.81
26	No Impairments	1.94	0.93
30	No Impairments	1.75	0.73
33	No Impairments	1.81	0.74
35	No Impairments	1.47	0.88
38	No Impairments	1.95	0.64
40	No Impairments	1.73	0.92
41	No Impairments	1.73	0.74
45	No Impairments	1.72	0.72

48	No Impairments	2.06	0.70
52	No Impairments	1.86	0.68
56	No Impairments	1.76	1.01
58	No Impairments	1.54	0.82
59	No Impairments	1.63	1.13
60	No Impairments	1.62	0.75
61	No Impairments	1.40	0.79
62	No Impairments	1.87	0.77
65	No Impairments	1.20	0.63
66	No Impairments	1.54	0.92
67	No Impairments	1.68	0.72
68	No Impairments	2.05	0.62
1	Combined	1.87	0.60
5	Pelvic	1.86	0.85
7	Thoracic	1.69	0.73
8	Combined	1.50	0.77
9	Combined	1.24	0.79
12	Pelvic	1.92	0.56
24	Pelvic	1.98	0.94
27	Pelvic	1.05	0.81
28	Pelvic	1.18	0.75
29	Pelvic	1.33	0.78
31	Combined	1.53	0.71
32	Thoracic	1.91	0.68
34	Combined	1.65	0.65
36	Thoracic	1.52	0.92
37	Thoracic	1.52	0.65
39	Pelvic	1.55	0.68
42	Pelvic	2.16	0.82
43	Pelvic	1.04	0.53
46	Thoracic	1.80	0.62
47	Pelvic	1.50	0.63
49	Pelvic	1.80	0.96
50	Combined	1.63	0.70
51	Thoracic	1.71	0.87
53	Pelvic	2.06	0.40
55	Thoracic	1.98	0.96
57	Thoracic	1.29	0.64
63	Pelvic	1.86	0.60
64	Pelvic	1.37	0.85
