# Supplementary material for: GenPup-M: A novel validated owner-reported clinical metrology instrument for detecting early mobility changes in dogs
Source: PLoS One. 2023 Dec 27;18(12):e0291035. doi: 10.1371/journal.pone.0291035 (PMC10752556; doi:10.1371/journal.pone.0291035)
Supplement: S4 Fig — (PDF) [file pone.0291035.s004.pdf]

Supporting Information 4. Kruskal-Wallis outputs from the comparison of body mass with normalised Peak Vertical Force (PVF) ratio in non-mobility impaired versus mobility impaired cohorts.

	**Kruskal-Wallis Peak Vertical Force (PVF) Output Means**
Thoracic Limbs	H (9.8) = 3, p = 0.021
Pelvic Limbs	H (13.8) = 3, p = 0.003
Thoracic to Pelvic Limbs Ratio	H (4.7) = 3, p = 0.019
