# Supplementary material for: GenPup-M: A novel validated owner-reported clinical metrology instrument for detecting early mobility changes in dogs
Source: PLoS One. 2023 Dec 27;18(12):e0291035. doi: 10.1371/journal.pone.0291035 (PMC10752556; doi:10.1371/journal.pone.0291035)
Supplement: S5 Fig — Highest score for the questionnaire was 108 and the severity was categorised as follows: score of 0–27 = No concerns; score of 28–54 = Mild concerns; score of 55–82 = Moderate concerns; score of 82–108 = Severe concerns. Questions 12–21 were used for the correlation statistics due to these being owner reported scale/rank questions relating to their dog’s activity levels, willingness to exercise and general demeanour. (PDF) [file pone.0291035.s005.pdf]

Supporting Information 5. The scoring system used for GenPup-M to create a total clinical examination score used for correlation of GenPup-M responses to LOAD questionnaire, clinical examination and Peak Vertical Force (PVF).

Question number	Number of points based on owner-reported responses
1	Very well = 0<br>Fairly well = 0<br>Satisfactory = 1<br>Poorly = 2<br>Very poorly = 3
1. a	I'm unsure = 0<br>Yes = 1<br>No = 0
1. b	I'm unsure = 0<br>Yes = 1<br>No = 0
2	No points assigned to this question
3	No points assigned to this question
4	No points assigned to this question
5	Never = 0<br>Yes, in the past = 1<br>Yes, currently receiving = 2<br>I don't know/can't remember = 0
7	No points assigned to this question
8	No points assigned to this question
9	No points assigned to this question
10	No points assigned to this question
11	No = 0<br>Only if looks tired/lame/stiff = 1<br>Walker limits exercise = 0<br>Other = 0
12	Not applicable = 0<br>**'0' on the scale = 0 points added to score**<br>'1' on the scale = 1 points added to score<br>'2' on the scale = 2 points added to score<br>'3' on the scale = 3 points added to score<br>'4' on the scale = 4 points added to score<br>'5' on the scale = 5 points added to score<br>'6' on the scale = 6 points added to score<br>'7' on the scale = 7 points added to score<br>'8' on the scale = 8 points added to score<br>'9' on the scale = 9 points added to score<br>'10' on the scale = 10 points added to score
13	Not applicable = 0<br>**'0' on the scale = 0 points added to score**<br>'1' on the scale = 1 points added to score<br>'2' on the scale = 2 points added to score<br>'3' on the scale = 3 points added to score

	‘4’ on the scale = 4 points added to score<br>‘5’ on the scale = 5 points added to score<br>‘6’ on the scale = 6 points added to score<br>‘7’ on the scale = 7 points added to score<br>‘8’ on the scale = 8 points added to score<br>‘9’ on the scale = 9 points added to score<br>‘10’ on the scale = 10 points added to score
14	Not applicable = 0<br>**‘10’ on the scale = 0 points added to score**<br>‘9’ on the scale = 1 points added to score<br>‘8’ on the scale = 2 points added to score<br>‘7’ on the scale = 3 points added to score<br>‘6’ on the scale = 4 points added to score<br>‘5’ on the scale = 5 points added to score<br>‘4’ on the scale = 6 points added to score<br>‘3’ on the scale = 7 points added to score<br>‘2’ on the scale = 8 points added to score<br>‘1’ on the scale = 9 points added to score<br>‘0’ on the scale = 10 points added to score
15	Not applicable = 0<br>**‘0’ on the scale = 0 points added to score**<br>‘1’ on the scale = 1 points added to score<br>‘2’ on the scale = 2 points added to score<br>‘3’ on the scale = 3 points added to score<br>‘4’ on the scale = 4 points added to score<br>‘5’ on the scale = 5 points added to score<br>‘6’ on the scale = 6 points added to score<br>‘7’ on the scale = 7 points added to score<br>‘8’ on the scale = 8 points added to score<br>‘9’ on the scale = 9 points added to score<br>‘10’ on the scale = 10 points added to score
16	Not applicable = 0<br>**‘10’ on the scale = 0 points added to score**<br>‘9’ on the scale = 1 points added to score<br>‘8’ on the scale = 2 points added to score<br>‘7’ on the scale = 3 points added to score<br>‘6’ on the scale = 4 points added to score<br>‘5’ on the scale = 5 points added to score<br>‘4’ on the scale = 6 points added to score<br>‘3’ on the scale = 7 points added to score<br>‘2’ on the scale = 8 points added to score<br>‘1’ on the scale = 9 points added to score<br>‘0’ on the scale = 10 points added to score
17	Not applicable = 0<br>**‘10’ on the scale = 0 points added to score**<br>‘9’ on the scale = 1 points added to score<br>‘8’ on the scale = 2 points added to score<br>‘7’ on the scale = 3 points added to score<br>‘6’ on the scale = 4 points added to score<br>‘5’ on the scale = 5 points added to score<br>‘4’ on the scale = 6 points added to score<br>‘3’ on the scale = 7 points added to score<br>‘2’ on the scale = 8 points added to score<br>‘1’ on the scale = 9 points added to score

	‘0’ on the scale = 10 points added to score
18	Not applicable = 0<br>**‘0’ on the scale = 0 points added to score**<br>‘1’ on the scale = 1 points added to score<br>‘2’ on the scale = 2 points added to score<br>‘3’ on the scale = 3 points added to score<br>‘4’ on the scale = 4 points added to score<br>‘5’ on the scale = 5 points added to score<br>‘6’ on the scale = 6 points added to score<br>‘7’ on the scale = 7 points added to score<br>‘8’ on the scale = 8 points added to score<br>‘9’ on the scale = 9 points added to score<br>‘10’ on the scale = 10 points added to score
19	Not applicable = 0<br>**‘0’ on the scale = 0 points added to score**<br>‘1’ on the scale = 1 points added to score<br>‘2’ on the scale = 2 points added to score<br>‘3’ on the scale = 3 points added to score<br>‘4’ on the scale = 4 points added to score<br>‘5’ on the scale = 5 points added to score<br>‘6’ on the scale = 6 points added to score<br>‘7’ on the scale = 7 points added to score<br>‘8’ on the scale = 8 points added to score<br>‘9’ on the scale = 9 points added to score<br>‘10’ on the scale = 10 points added to score
20	Not applicable = 0<br>**‘0’ on the scale = 0 points added to score**<br>‘1’ on the scale = 1 points added to score<br>‘2’ on the scale = 2 points added to score<br>‘3’ on the scale = 3 points added to score<br>‘4’ on the scale = 4 points added to score<br>‘5’ on the scale = 5 points added to score<br>‘6’ on the scale = 6 points added to score<br>‘7’ on the scale = 7 points added to score<br>‘8’ on the scale = 8 points added to score<br>‘9’ on the scale = 9 points added to score<br>‘10’ on the scale = 10 points added to score
21	Not applicable = 0<br>**‘0’ on the scale = 0 points added to score**<br>‘1’ on the scale = 1 points added to score<br>‘2’ on the scale = 2 points added to score<br>‘3’ on the scale = 3 points added to score<br>‘4’ on the scale = 4 points added to score<br>‘5’ on the scale = 5 points added to score<br>‘6’ on the scale = 6 points added to score<br>‘7’ on the scale = 7 points added to score<br>‘8’ on the scale = 8 points added to score<br>‘9’ on the scale = 9 points added to score<br>‘10’ on the scale = 10 points added to score
22	No points assigned to this question

Highest score for the questionnaire was 108 and the severity was categorised as follows:

- Score of 0 – 27 = No concerns
- Score of 28 – 54 = Mild concerns
- Score of 55 – 82 = Moderate concerns
- Score of 82 – 108 = Severe concerns

Questions 12-21 were used for the correlation statistics due to these being owner-reported scale/rank questions relating to their dog's activity levels, willingness to exercise and general demeanour.
